# *Citrus sinensis* MYB Transcription Factor CsMYB85 Induce Fruit Juice Sac Lignification Through Interaction With Other CsMYB Transcription Factors

**DOI:** 10.3389/fpls.2019.00213

**Published:** 2019-02-25

**Authors:** Ning Jia, Jiqin Liu, Penghui Tan, Yufeng Sun, Yuemeng Lv, Jiameng Liu, Jing Sun, Yatao Huang, Jia Lu, Nuo Jin, Minmin Li, Khandaker Md Sharif Uddin Imam, Fengjiao Xin, Bei Fan

**Affiliations:** ^1^Key Laboratory of Agro-Products Quality and Safety Control in Storage and Transport Process, Ministry of Agriculture and Rural Affairs, Institute of Food Science and Technology, Chinese Academy of Agricultural Sciences, Beijing, China; ^2^Laboratory of Quality and Safety Risk Assessment on Agro-Products Processing, Ministry of Agriculture and Rural Affairs, Beijing, China; ^3^Laboratory of Biomanufacturing and Food Engineering, Institute of Food Science and Technology, Chinese Academy of Agricultural Sciences, Beijing, China; ^4^Qinhuangdao Customs, Hebei Qinhuangdao, Qinhuangdao, China; ^5^Turfgrass Research Institute, Beijing Forestry University, Beijing, China

**Keywords:** *Citrus sinensis*, CsMYB85, juice sacs, lignification, postharvest

## Abstract

Varieties of *Citrus* are commercially important fruits that are cultivated worldwide and are valued for being highly nutritious and having an appealing flavor. Lignification of citrus fruit juice sacs is a serious physiological disorder that occurs during postharvest storage, for which the underlying transcriptional regulatory mechanisms remain unclear. In this study, we identified and isolated a candidate MYB transcription factor, CsMYB85, that is involved in the regulation of lignin biosynthesis in *Citrus sinensis*, which has homologs in *Arabidopsis* and other plants. We found that during juice sac lignification, *CsMYB85* expression levels increase significantly, and therefore, suspected that this gene may control lignin biosynthesis during the lignification process. Our results indicated that CsMYB85 binds the *CsMYB330* promoter, regulates its expression, and interacts with CsMYB308 in transgenic yeast and tobacco. A transient expression assay indicated that *Cs4CL1* expression levels and lignin content significantly increased in fruit juice sacs overexpressing CsMYB85. *At4CL1* expression levels and lignin content were also significantly increased in *Arabidopsis* overexpressing CsMYB85. We accordingly present convincing evidence for the participation of the CsMYB85 transcription factor in fruit juice sac lignification, and thereby provide new insights into the transcriptional regulation of this process in citrus fruits.

## Introduction

Varieties of *Citrus* are commercially important fruits cultivated worldwide that are valued for being highly nutritious and having an appealing flavor. Citrus fruit juice sac granulation (lignification) is a serious physiological disorder that occurs during the postharvest storage of these fruits (Shomer et al., 1989). In the granulation process, the pulp tissue becomes hard and lignified, which diminishes fruit nutritional and commercial value (Pan et al., 1999). As fruit juice sac granulation increases, the cell walls thicken by lignification. Consequently, the juice sacs become deformed and atrophied, and the number of normal juice cells gradually decreases (Shomer et al., 1989; Pan et al., 1999). Previous studies have demonstrated that juice sac lignin content increases during granulation and plays a vital role in this process (Zhang et al., 2016; Jia et al., 2018). Lignin accumulation is significantly correlated with the juice sac granulation index (Zhang et al., 2016). During the granulation process, transcription levels of the *CmMYB330* and *CsMYB308* transcription factor genes are either significantly upregulated or downregulated and homologs identified in *Arabidopsis* and other plant species have also been identified as being involved in lignin biosynthesis regulation (Yang et al., 2017a; Jia et al., 2018). Accordingly, an in-depth functional study of lignin biosynthesis-related genes may enhance our understanding of juice sac granulation.

The biosynthesis of lignin involves a series of sequential enzymatic reactions, in the first step of which, cinnamic acid hydroxylase (C4H), phenylalanine ammonia-lyase (PAL), hydroxystyrene CoA, and ligase (4CL) participate in the phenylpropylene ring pathway. Thereafter, caffeic acid-*O*-methyltransferase (COMT) and ferulate 5-hydroxylase (F5H) methylate lignin monomers, which are further modified via cinnamyl alcohol dehydrogenase (CAD) and cinnamate-CoA reductase (CCR) (Kawaoka et al., 2010; Vanholme et al., 2010), followed by polymerization catalyzed by PER/LAC.

A number of MYB and NAC transcription factors play important roles in the transcription and regulation of lignin synthesis-related genes (Zhong et al., 2006; Golfier et al., 2017; Koshiba et al., 2017), as both transcriptional activators and repressors. Previously, it has been demonstrated that overexpression of *AtMYB46* or *AtMYB83* promotes the upregulation of lignin synthesis-related genes, resulting in abnormal secondary cell wall accumulation (Ko et al., 2009). In the *Arabidopsis* *myb46myb83* double mutant, plants are unable to form secondary walls and die during the seedling stage (Wang and Dixon, 2012). In other plant species, several MYB transcription factors are associated with lignin synthesis, including *PtrMYB3*, *PtrMYB20*, *EgMYB2*, *PvMYB4*, *PtMYB1*, *PtMYB4*, *PtMYB8*, *OsMYB46*, and *ZmMYB* (Patzlaff et al., 2003; Bomal et al., 2008; McCarthy et al., 2010; Rahantamalala et al., 2010; Zhong et al., 2011; Shen et al., 2012). In tomato, SlMYB1 and SlMYB2 have been found to regulate cellulose metabolism (Shi et al., 2017), whereas in chrysanthemum, overexpression of the *CmMYB19* transcription enhances lignin content and aphid tolerance in transgenic plants (Wang et al., 2017). Numerous MYB transcription factors, including AtMYB58, AtMYB63, and AtMYB85, recognize and bind lignin synthesis gene promoters, thereby regulating their expression (Zhong et al., 2007; Zhou et al., 2009).

Transcriptional repressors that have been implicated in lignin synthesis include *Arabidopsis* AtMYB32 and AtMYB4 (Jin et al., 2000; Preston et al., 2004). Overexpression of AtMYB4 in tobacco results in a significant downregulation of lignin synthesis-related gene expression and retards growth (Jin et al., 2000), whereas AtMYB32 binds to the *COMT* promoter and upregulates *COMT* expression in the *myb32* mutant (Preston et al., 2004). Several transcriptional repressors have also been identified in other plants, including eucalyptus (*EgMYB1*) (Legay, 2005), Antirrhinum (*AmMYB330* and *AmMYB308*) (Tamagnone et al., 1998), *Eriobotrya japonica* (*EjMYB2*) (Xu et al., 2014), and maize (*ZmMYB31* and *ZmMYB42*) (Sonbol et al., 2009; Fornalé et al., 2010). EgMYB1 downregulates *EgCCR* and *EgCAD* expression (Legay et al., 2007), whereas PtoMYB156 downregulates secondary wall formation during xylogenesis in *Populus tomentosa* via the phenylpropanoid pathway (Yang et al., 2017b), and overexpression of Maize *ZmMYB31* in *Arabidopsis thaliana* reduces lignin content of transgenic plants, which show a dwarf phenotype (Fornalé et al., 2010).

Aspects of the regulatory mechanisms underlying fruit lignification have been reported previously. In *Citrus sinensis*, the CsMYB330 and CsMYB308 transcription factors have been found to regulate fruit juice sac lignification by regulating the expression of *Cs4CL1* (Jia et al., 2018). Loquat fruit lignin content significantly increases following cold exposure, and in this context, and it has been shown that EjMYB1 and EjMYB2 recognize and bind to the AC element of the *Ej4CL1* promoter, thereby regulating *Ej4CL1* expression and lignin biosynthesis (Xu et al., 2014). EjMYB8 has also been found to regulate lignin biosynthesis in loquat fruits (Wang et al., 2016) and EjNAC3 regulates *EjCAD-like* expression and influences lignin content (Ge et al., 2017). Although EjAP2-1 is known to regulate lignin biosynthesis in loquat fruits, it does not directly influence lignin synthesis gene expression, but instead controls interactions between the EjMYB1 and EjMYB2 proteins (Zeng et al., 2015).

Nevertheless, although a number of MYB transcription factors related to lignin metabolism have been widely studied, their functions in citrus fruit lignification have seldom been analyzed. In this study, we isolated and identified the R2R3 MYB transcription factor CsMYB85 and examined its roles in citrus fruits juice sac lignification. Our results indicate that this CsMYB transcription factor indirectly regulates citrus fruit juice sac lignification.

## Materials and Methods

### Plant Materials and Growth Conditions

In June 2017, fresh naturally ripened *Citrus sinensis* fruits of similar size and maturity were harvested and selected in Yichang, Hubei Province, China. They were maintained in ventilated cold storage in which the climate was controlled at 8.0 ± 2.0°C and 75.5% ± 5.0% relative humidity (RH). The fruit juice sacs showed different levels of granulation according to the method described by Zhang et al. (2016).

Wild-type (WT) *Arabidopsis* *thaliana* (Columbia) and *Nicotiana benthamiana* were grown either in perlite/vermiculite/peat soil (1:1:1 v/v/v) or on Murashige and Skoog (MS) media plates in a greenhouse under 16 h light and 8 h darkness at 22°C and 40–60% RH.

### RNA and DNA Extraction and cDNA Synthesis

Total RNA and genomic DNA of *C sinensis* fruit juice sacs and *Arabidopsis* plants were extracted using RNA and DNA extraction kits (Aidlab, Beijing, China). The cDNA was synthesized from 2.5 μg of total RNA using a RevertAid premium first-strand cDNA synthesis kit (Fermentas, Thermo Fisher Scientific, Rochester, NY, United States), and used as a template for quantitative real-time polymerase chain reaction (qRT-PCR) and reverse-transcription PCR (RT-PCR) analyses. For qRT-PCR, we used a SYBR Green PCR MasterMix (TaKaRa Bio, Inc., Kusatsu, Shiga, Japan). Amplification was performed using a 7500 fast quantitative PCR system (Applied Biosystems, Foster City, CA, United States). *CsActin* and *AtActin* were used as reference genes to normalize expression levels. All qRT-PCR analyses were repeated on four biological replicates. The primers used for amplification are listed in Supplementary Table 1.

### Gene Cloning and Sequence Analysis

On the basis of the sweet orange genome sequence (Xu et al., 2013), the CsMYB85 coding region was cloned via RT-PCR using gene-specific primers (Supplementary Table 2). The promoters were isolated by PCR using the promoter-specific primers listed in Supplementary Table 2. The RT-PCR products were cloned in the SK vector. The protein sequences were blasted in the NCBI database to identify homologous sequences in other plant species. DNAMAN v. 6.0 (Lynnon LLC, San Ramon, CA, United States) was used to align and construct a phylogenetic tree of MYB proteins.

### Subcellular Localization Analysis

The subcellular localization of CsMYB85 was determined by cloning the full-length coding region lacking a stop codon into the 3302GFP vector fused with the green fluorescent protein (GFP) gene. *Pro-35S::CsMYB85-GFP* and control 3302GFP (*Pro-35S::GFP*) vectors were transfected into *Agrobacterium* (GV3101) and subsequently transiently expressed in *N. benthamiana* leaf cells (Voinnet et al., 2003). After infiltration for 48 h, fluorescence images were observed under a confocal laser scanning microscope (SP8; Leica Microsystems, Wetzlar, Germany). Cell nuclei were stained with 4’,6-diamidino-2- phenylindole (DAPI). The primers used for cloning are listed in Supplementary Table 1.

### Lignin Content Analysis

Lignin content was determined using the method described by Zhang et al. (2016). All lignin content analyses were performed with three biological replicates.

### Yeast Assay

To assess CsMYB85 transcriptional activation in yeast cells, *CsMYB85* was cloned into the pGBKT7 (BD) vector (Clontech Laboratories, Mountain View, CA, United States). BD-CsMYB85, pGADT7 (AD), and a negative control (AD + BD vector) were transformed into the yeast strain Y2H Gold (Clontech Laboratories, Mountain View, CA, United States) using PEG/LiAc method. The yeast cells were grown on SD medium lacking Leu and Trp (SD/-Leu-Trp or SD/-L-T) or Leu, Trp, and His (SD/-Leu-Trp-His or SD/-L-T-H). Transcriptional activation by CsMYB85 was evaluated according to yeast growth status.

*CsMYB85* coding sequences were ligated into to the AD vector, whereas the *CsMYB330* and *CsMYB308* promoters were inserted into the pHIS2 vector. The primers used for vector construction are listed in Supplementary Table 2. Other vectors used have been described previously (Jia et al., 2018). Recombinant pHIS2 and empty AD (control) vectors were transformed into yeast strain Y187 (Clontech Laboratories, Mountain View, CA, United States), which was grown on SD screening medium (SD/-Leu-Trp-His) containing different concentrations of 3-amino-1,2,4-triazole (3-AT), an appropriate concentration of which suppresses background histidine leakiness in pHIS2 vectors. Interactions between CsMYB85 and its promoters were assessed on SD screening medium (SD/-Leu-Trp-His) containing the optimal 3-AT concentration.

The coding sequences of CsMYB85 and CsMYB308 were inserted into the pGADT7 (AD) and pGBKT7 (BD) vectors. BD-53 and AD-T were used as positive controls and empty BD and AD were used as negative controls. The plasmids were co-transfected into yeast strain AH109 (Clontech Laboratories, Mountain View, CA, United States) via PEG-mediated transformation, and the transformed yeast cells were grown on SD screening medium (SD/-Leu-Trp and SD/-Leu-Trp-His) for 3 days at 30°C.

### Transcriptional Activity Assay

On the basis of the results of the one-hybrid yeast assay, transcriptional activity was analyzed according to Liu et al. (2016) and Jia et al. (2018). The *CsMYB330* promoter was cloned into the 35S-LUC-GUS vector to drive *GUS* reporter gene expression, with *LUC* being used as an internal standard. The coding sequence of the CsMYB85 transcription factor was cloned into the 35S-LUC-GUS vector driven by the cauliflower mosaic virus 35S (CAM35S) promoter. The plasmids were transformed into *Agrobacterium* GV3101, which was cultured in infiltration buffer containing 10 mM MgCl_2_, 10 mM MES, and 100 μM acetosyringone until OD_600_ = 0.5. For transcriptional activity analysis, *N. benthamiana* plants were infiltrated with *Agrobacterium* GV3101 containing recombinant plasmids using needleless syringes. GUS and LUC activities were measured after 3 days. The primers used for vector construction are listed in Supplementary Table 2.

### Bimolecular Fluorescence Complementation (BiFC)

BiFC assays were performed using *N. benthamiana* leaves as described previously (Voinnet et al., 2003). CsMYB85 and CsMYB308 sequences were inserted into 35S-SPYNE and 35S-SPYCE vectors and fused with *N*-terminal yellow fluorescent protein (YFP^N^) and/or *C*-terminal YFP (YFP^C^). The BiFC vectors were introduced into *Agrobacterium* GV3101, which was infiltrated into *N. benthamiana* leaves using a needleless syringe. YFP fluorescence signals were visualized under a confocal laser scanning microscope (SP8; Leica Microsystems, Wetzlar, Germany) 3 days after infiltration. The primers used for BiFC vector construction are listed in Supplementary Table 2.

### Transient Juice Sac Assay by Particle Gun Bombardment

Juice sacs were separated from sweet orange fruits and placed into a Petri dish containing MS medium supplemented with 0.2% gelrite (Endo et al., 2007). The coding sequence of *CsMYB85* sequence was inserted into the SK vector under control of the CAM35S promoter. Empty SK vector was used as a control. The PDS-1000/He particle delivery system (Bio-Rad Laboratories, Hercules, CA, United States) was used for particle gun bombardment according to manufacturer instructions. Gold particles (1.0 μm in diameter) were packed with SK-CsMYB85 or SK plasmids. The target distance between the Petri dish and the stop screen was 9 cm and helium pressure was 9.3 MPa. After bombardment, the juice sacs were incubated on MS medium at 25°C and subsequently collected at 24 and 120 h for gene expression analysis and lignin content assay, respectively.

## Results

### CsMYB85 Isolation and Analysis

**FIGURE 1:**
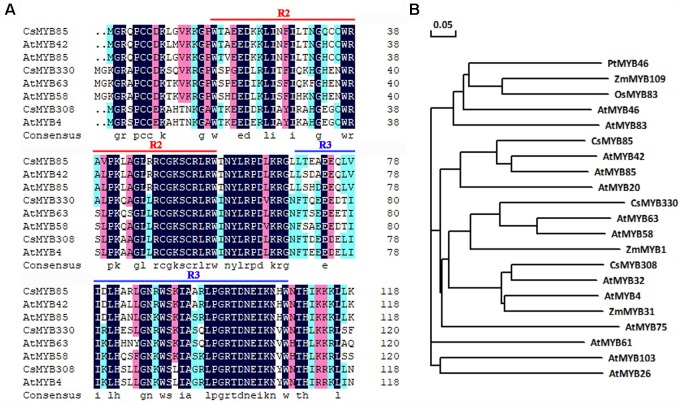
CsMYB85 sequence and phylogenetic analysis. **(A)** Alignment of the conserved R2R3 domains in CsMYB85 with those in AtMYB42, AtMYB85, AtMYB58, AtMYB63, AtMYB4, CsMYB330, and CsMYB308. **(B)** Phylogenetic tree for CsMYB85 and MYB transcription factors from other plants: *Populus trichocarpa*, *Arabidopsis thaliana*, *Zea mays*, and *Oryza sativa*. The accession numbers of the transcription factor proteins are as follows: PtMYB46 (XP_024465368.1), ZmMYB109 (NP_001241859.1), OsMYB83 (XP_015619488.1), AtMYB46 (AT5G12870), AtMYB83 (At3g08500), CsMYB85 (XP_006477265.1), AtMYB42 (AT4G12350), AtMYB85 (AT4G22680), AtMYB20 (AT4G66230), CsMYB330 (NC_023049), AtMYB63 (AT1G79180), AtMYB58 (AT1G16490), ZmMYB1 (P20024.1), CsMYB308 (NC_023053), AtMYB32 (AT4G34990), AtMYB4 (AT4G38620), ZmMYB31 (NP_001105949), AtMYB75 (AT1G56650), AtMYB61 (AT1G09540), AtMYB103 (AT1G63910), and AtMYB26 (AT3G13890) The phylogenetic tree was constructed using DNAMAN v. 6.0.

*CsMYB85* isolated from sweet orange (*C. sinensis*) and its orthologs in other plants play important roles in lignin biosynthesis. The amino acid sequence of this transcription factor is similar to that of AtMYB85 from *Arabidopsis* (60%), and was accordingly named *CsMYB85*. *CsMYB85* encodes a protein comprising 267 amino acids and has an isoelectric point and molecular weight of 5.08 and 30.14 kDa, respectively. CsMYB85 has a conserved R2R3 domain and is thus classified as an R2R3 MYB transcription factor (Figure 1A). In a phylogenetic tree constructed for MYB transcription factors, we found that CsMYB85 was clustered with the lignin-related AtMYB85 and AtMYB42 (Figure 1B), which may also regulate lignin biosynthesis (Zhong et al., 2008). We accordingly speculate that CsMYB85 is involved in the regulation of lignin biosynthesis.

### *CsMYB85* Expression in Citrus Fruits at Different Stages of Granulation

**FIGURE 2:**
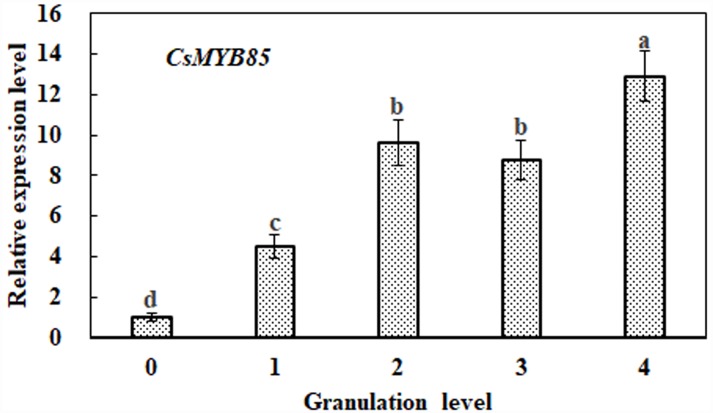
Relative expression of the *CsMYB85* gene at different stages of citrus fruit granulation. *CsMYB85* expression was determined by real-time qRT-PCR and normalized to the *CsActin* reference gene. Means were obtained from four biological replicates. Error bars represent standard errors and were determined using Duncan’s multiple range test (α = 0.05) in SAS software (SAS Institute, Cary, NC, United States). The ‘a–d’ above each column indicates *P* < 0.05 and the same letter indicates that the difference is not significant.

In previous studies, it has been observed that lignin content increases significantly with the progression of citrus fruit granulation (Zhang et al., 2016; Jia et al., 2018). On the basis of *CsMYB85* expression during citrus fruit granulation, we performed qRT-PCR analysis to determine its association with lignin biosynthesis. We accordingly found that *CsMYB85* transcription levels increased with the advance of citrus fruit granulation, being relatively low at granulation level 0, but showing an approximate 13-fold increase by level 4 (Figure 2). This increase in the expression level *CsMYB85* is consistent with the increase in lignin content during citrus fruit granulation (Figure 2).

### Nuclear Localization and Transcriptional Activation of CsMYB85

**FIGURE 3:**
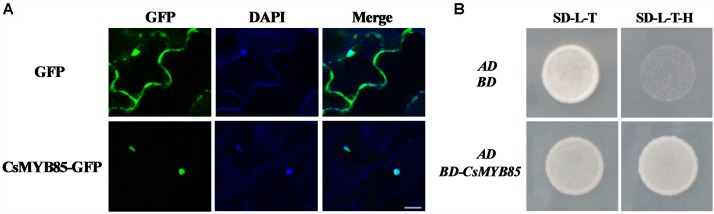
Subcellular localization and transcriptional activation of CsMYB85. **(A)** Subcellular localization of CsMYB85 in tobacco leaf cells. 35S:GFP was localized in both the nucleus and cytoplasm of tobacco leaf cells. In contrast, CsMYB330:GFP was observed only in the nucleus. 4’,6-Diamidino-2-phenylindole (DAPI) signals were localized only in the nucleus. Merged images show GFP and DAPI colocalization. Bar = 20 μm. **(B)** Transcriptional activation of CsMYB85 in yeast cells. The full-length coding sequence of CsMYB85 was inserted into the pGBKT7 (BD) vector. The pGADT7 (AD) vector and either BD-CsMYB85 or BD were transformed into cells of the Y2HGold yeast strain. Yeast cells containing AD and BD vectors were used as negative controls. The yeast was grown on SD media lacking –Leu/–Trp (–L/–T) or –Leu/–Trp/–His (–L/–T/–H) for 3 days at 30°C. Results were obtained from three independent transformation experiments.

MYBs transcription factors are typically nuclear proteins (Zhong et al., 2007; Xu et al., 2014; Jia et al., 2018). Consistently, we found that when a vector harboring CsMYB85 and GFP under control of the CaMV 35S promoter was expressed in tobacco leaves, CsMYB85-GFP signals were detected in the nucleus, whereas positive control GFP signals were observed in both nucleus and cytoplasm (Figure 3A). To examine CsMYB85 transcriptional activity, a pGBKT7 (BD)-CsMYB85 recombinant vector was constructed and transformed into the Y2HGold strain of yeast along with pGADT7 (AD). We observed that the pGADT7 (AD) vector could activate Leu synthase and the pGBKT7 (BD) vector containing CsMYB85 could activate Trp and His synthase, and thus defective Y2HGold yeast containing the BD-CsMYB85 and AD vectors grew well on SD medium lacking Leu/Trp/His (–L/–T/–H) medium for 3 days at 30°C (Figure 3B), thereby indicating that CsMYB85 is transcriptionally active in yeast.

### *CsMYB85* Binds to Lignin Biosynthesis-Related Gene Promoters and Regulates *CsMYB330* Expression

**FIGURE 4:**
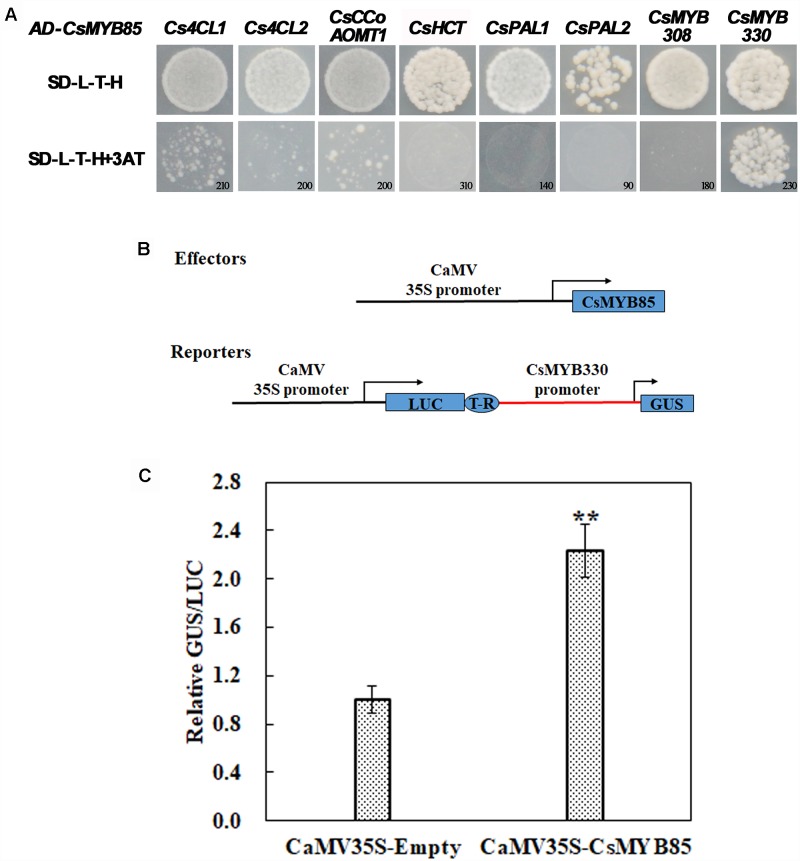
Interactions between CsMYB85 and the promoters of lignin biosynthesis-related genes. **(A)** Interactions of CsMYB85 with the promoters of *CsMYB330*, *CsMYB308*, *Cs4CL1*, *Cs4CL2*, *Cs4CCoAOMT1*, *CsHCT*, *CsPAL1*, and *CsPAL2* were identified using Y1H assays. The promoters were inserted into pHIS2 vectors. Empty AD and constructed pHIS2 vectors were transfected into yeast Y187 cells, which were grown on SD/–L/–T/–H dropout medium containing various concentrations of 3-amino-1,2,4-triazole (3-AT) to suppress background histidine leakiness. Numbers in the lower-right corner of the images indicate the optimal 3-AT concentrations. Results were obtained from three independent transformation experiments. **(B)** Activation of the *CsMYB330* promoter by CsMYB85 was assayed transiently in *Nicotiana benthamiana* leaves using an effector and reporter system. The effector and reporter constructs are shown in the schematic diagram. The reporter vector contained *LUC* normalization and *GUS* reporter genes driven by the *CaMV35S* and *CsMYB330* promoters, respectively. The effector vector contained a *CsMYB85* gene under control of the *CaMV35S* promoter. T-R, terminator; Boxes, various DNA sequences. **(C)** Transcriptional activity of CsMYB85 was analyzed using a *GUS* reporter gene driven by the *CsMYB330* promoter. The GUS/LUC ratio in leaves transformed with the empty vector (control) harboring the *CsMYB330* promoter was set to 1. Error bars represent standard errors. Means were obtained from five biological replicates. Student’s *t*-test: ^∗∗^*P* < 0.01.

To confirm the interaction between CsMYB85 and lignin synthesis-related genes, their promoter fragments were cloned from citrus fruit genomic DNA. To establish whether CsMYB85 binds directly to the promoters of these genes, we used the yeast one-hybrid (Y1H) system. The constructed pHIS2 and empty AD vectors were transformed into yeast strain Y187, which was subsequently cultured on screening medium (SD/-Leu-Trp-His) containing different concentrations of 3-AT ranging from 0 to 250 mM. The Y1H assay indicated that CsMYB85 interacts directly and exclusively with the *CsMYB330* promoter to activate its expression (Figure 4A), and therefore the CsMYB330 promoter was used in subsequent experiments.

We cloned the *CsMYB330* promoter, which we used to drive the GUS reporter gene, and *CsMYB85* was driven by the CaMV35S promoter (Figure 4B). *Agrobacterium* (GV3101) cells containing *35S::CsMYB85* and *ProCsMYB330::GUS* plasmids were used to transform tobacco plants for transient expression analysis, and the results showed that CsMYB85 activated expression of the *GUS* reporter gene driven by the *CsMYB330* promoter in tobacco leaves (Figure 4C). Thus, we deduced that CsMYB85 activates *CsMYB330* expression.

### Protein–Protein Interactions Between CsMYB85 and the Juice Sac Lignification-Related Transcription Factor CsMYB308

**FIGURE 5:**
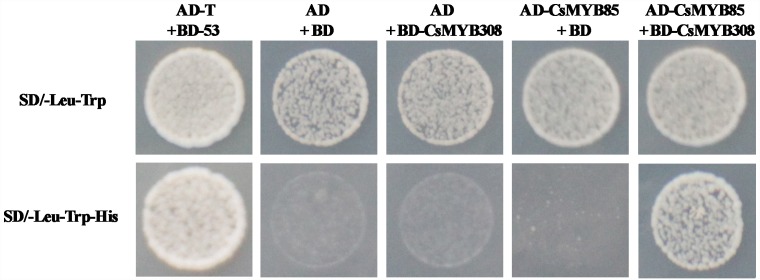
Interaction between CsMYB85 and CsMYB308 determined using a yeast two-hybrid assay. Analyses of CsMYB85 and CsMYB308 interactions in yeast cells. The CsMYB85 and CsMYB308 coding regions were cloned into the AD and BD vectors, respectively. AD-T containing BD-53 and AD containing BD were used as positive and negative controls, respectively. Yeast clones were grown on SD media lacking –Leu/–Trp or –Leu/–Trp/–His for 3 days at 30°C. Results were obtained from three independent transformation experiments.

The results of our previous study indicated that the CsMYB308 transcription factor binds directly binds to the *Cs4CL1* promoter in yeast cells and participates in juice sac lignification (Jia et al., 2018). However, CsMYB85 does not bind directly bind to lignin biosynthesis-related gene promoters. Therefore, we assumed that CsMYB85 may interact with other CsMYB transcription factors in lignin biosynthesis. To examine protein–protein interactions between CsMYB85 and CsMYB308, we used the yeast two-hybrid system. The coding sequences of CsMYB308 and CsMYB85 were inserted into the AD and BD vectors, respectively, and we found that whereas BD-CsMYB308 was not auto-activated in yeast (Jia et al., 2018), auto-activation was observed with BD-CsMYB85 (Figure 3B). Co-transfection of cells of the yeast strain AH109 with the BD-CsMYB308 and AD-CsMYB85 vectors indicates that CsMYB85 interacts with CsMYB308 (Figure 5).

**FIGURE 6:**
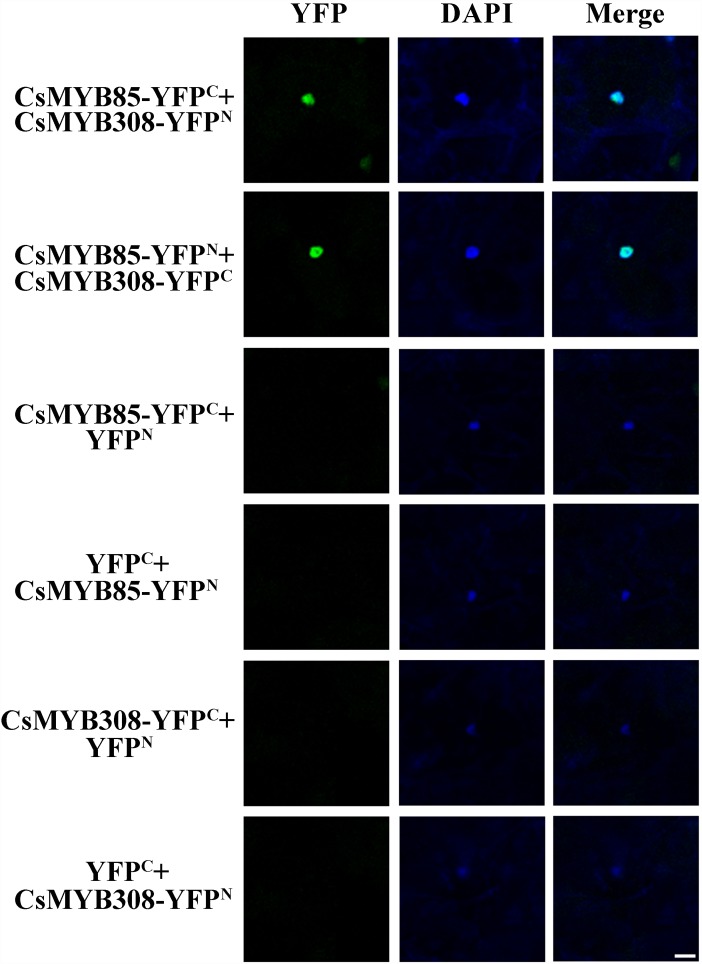
Interaction between CsMYB85 and CsMYB308 determined using a BiFC assay. CsMYB85 and CsMYB308 were fused with *C*-terminal yellow fluorescent protein (YFP^C^) and *N*-terminal YFP (YFP^N^), respectively, and driven by the *CaMV35S* promoter. The vectors were introduced into *Nicotiana benthamiana* leaves by agroinfiltration. YFP fluorescent signals were imaged under a confocal microscope 72 h after infiltration. Panels from left to right: YFP signal (YFP), 4′,6-diamidino-2-phenylindole (DAPI) nuclear staining signal, and merged YFP and DAPI signals. Empty vectors (YFP^C^ and YFP^N^) and the aforementioned vectors were co-expressed in epidermal cells as a control. Bar = 50 μm.

To validate the results of the yeast two-hybrid assays, BiFC was used to analyze the interaction between CsMYB85 and CsMYB308. These two transcription factors were fused with *N*-terminal YFP (YFP^N^) and/or *C*-terminal YFP (YFP^C^) driven by the *CaMV35S* promoter, and the fusion proteins were expressed in *N. benthamiana* leaves via *Agrobacterium* (GV3101)-mediated infiltration. A YFP fluorescent signal was detected in the epidermal cell nuclei of plants transformed with 35S:CsMYB85-YFP^N^, 35S:CsMYB308-YFP^C^/35S:CsMYB85-YFP^C^, and 35S:CsMYB308-YFP^N^ constructs, indicating that the CsMYB85 fusion proteins interact with CsMYB308 (Figure 6), thereby confirming the results obtained using the yeast two-hybrid assay.

### CsMYB85 Indirectly Regulates Cs4CL1 Expression *via* CsMYB330 and Affects Lignin Synthesis in Juice Sacs

**FIGURE 7:**
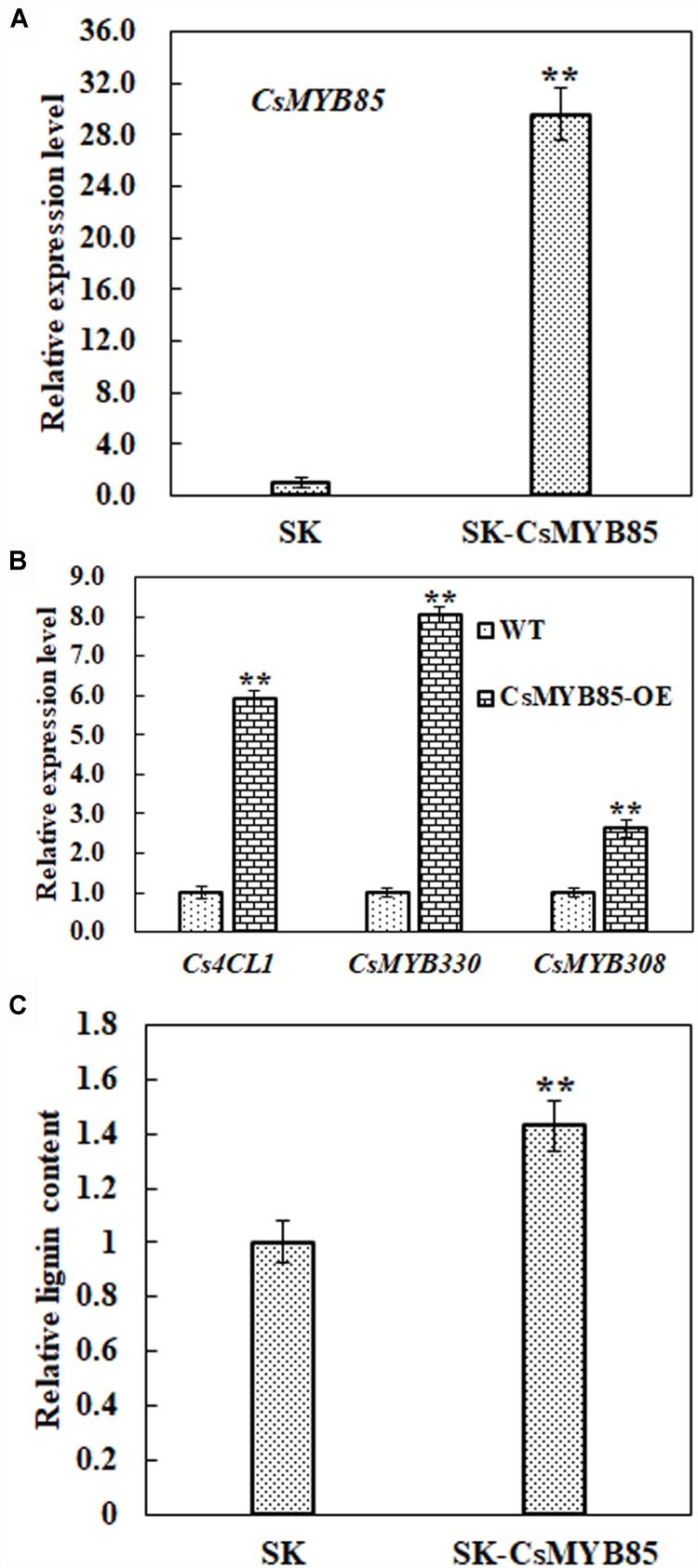
Lignin content and gene expression analyses in transiently overexpressing juice sacs. **(A)** Expression levels of the *CsMYB85* gene in transiently overexpressing juice sac tissues were determined using real-time qRT-PCR. Empty SK vector was used as a control. The *CsMYB85* expression level was set to 1 in the cintrol. Expression of the *CsActin* reference gene (*NW_006256915.1*) was used to normalize the *CsMYB85* expression level. Error bars represent the standard error of four biological replicates. Student’s *t*-test: ^∗∗^*P* < 0.01. **(B)** Transcription levels of *CsMYB330*, *CsMYB308*, and *Cs4CL1* (*NW_006257196.1*) in transiently overexpressing juice sac tissues determined by real-time qRT-PCR and normalized to the *CsActin* reference gene. Expression levels of *CsMYB330*, *CsMYB308*, and *Cs4CL1* in the SK control were set to 1. Error bars represent the standard error of four biological replicates. Student’s *t*-test: ^∗∗^*P* < 0.01. **(C)** Lignin content in juice sac tissues. Means represent the averages of three replicate lignin content determinations in transiently overexpressing juice sac tissues. Error bars represent standard errors. Student’s *t*-test: ^∗∗^*P* < 0.01.

Obtaining transgenic *C. sinensis* lines is typically laborious and time-consuming, and therefore we investigated the function of the CsMYB85 transcription factor in lignin biosynthesis regulation via a transient juice sac assay based on particle gun bombardment. We transiently overexpressed *CsMYB85* driven by the *CaMV35S* promoter in juice sac tissue and subsequently analyzed gene expression and lignin content. As shown in Figure 7A, CsMYB85 expression was significantly higher in plants overexpressing *CsMYB85* than in those transformed with the empty SK control. Figure 4, 5 indicate that CsMYB85 binds the *CsMYB330* promoter and regulates its expression in yeast and tobacco leaf cells. We performed a real-time qRT-PCR assay to evaluate the expression levels of *CsMYB330*, *CsMYB308*, and *Cs4CL1* in cells containing the SK and SK-CsMYB85 constructs, and the results indicated that the expression levels of all three genes were upregulated in transiently overexpressing juice sac tissue (Figure 7B). We found that the lignin content of juice sac tissue transiently overexpressing SK-CsMYB85 was approximately 1.4-fold higher than in tissue harboring the empty SK vector (Figure 7C). These results accordingly indicate that CsMYB85 indirectly regulates lignin biosynthesis.

### *CsMYB85* Overexpression Increases Lignin Content in *Arabidopsis thaliana*

**FIGURE 8:**
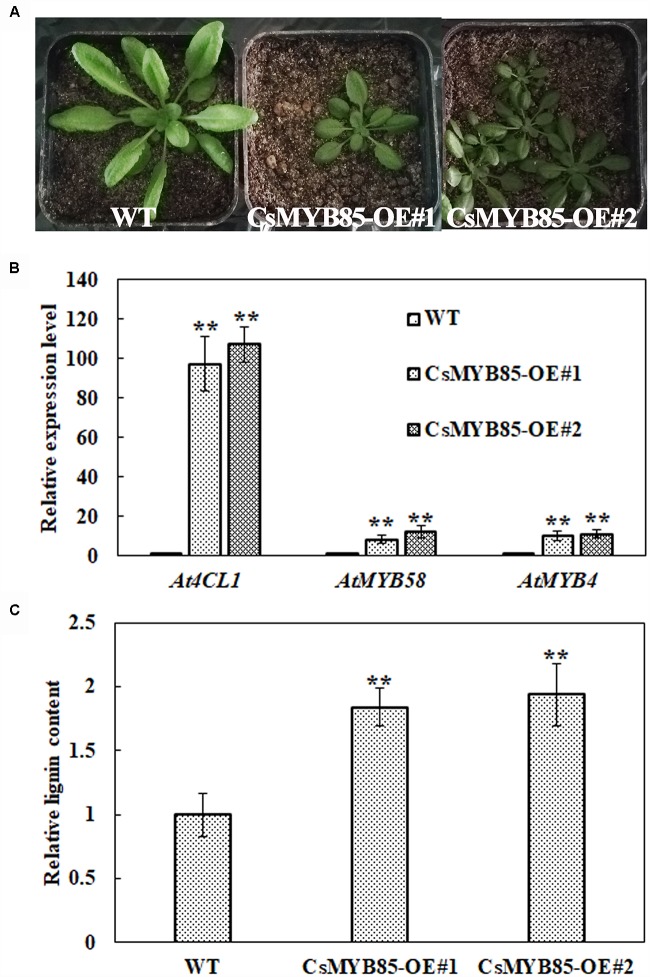
CsMYB85 overexpression increased lignin content in *Arabidopsis thaliana.* **(A)** Six-week-old seedlings of wild-type *A. thaliana* (WT, left), MYB85-OE#1 (CsMYB85 overexpression line 1, center), and MYB85-OE#2 (CsMYB85 overexpression line 2, right). Note the smaller leaves of MYB85-OE#1 and MYB85-OE#2 plants relative to those of the WT. **(B)** Expression levels of *At4CL1* (*AT1G51680*), *AtMYB58*, and *AtMYB4* were increased in CsMYB85-OE#1 and MYB85-OE#2 plants compared with those in WT plants. *At4CL1*, *AtMYB58*, and *AtMYB4* expression levels in the WT were set to 1. *AtActin* was used as an internal reference gene. Error bars represent the standard errors of four biological replicates. Student’s *t*-test: ^∗∗^*P* < 0.01. **(C)** Lignin content in WT, CsMYB85-OE#1, and MYB85-OE#2 plants. Data are the averages of four biological replicates in WT, CsMYB85-OE#1, and MYB85-OE#2 plants. Error bars represent standard errors. Student’s *t*-test: ^∗∗^*P* < 0.01.

We overexpressed *CsMYB85* under the control of the CaMV35S promoter in wild-type (WT) *Arabidopsis* (Figure 8) and evaluated gene expression and lignin content in the transgenic plants. We accordingly obtained nine CsMYB85 overexpression (CsMYB85-OE) lines and subsequently analyzed overexpression lines 1 and 2, which were characterized by smaller rosettes than those of WT plants (Figure 8A). The *Arabidopsis* genes *AtMYB58* and *AtMYB4* are orthologous to the *CsMYB330* and *CsMYB308* genes, respectively, in *C. sinensis* (Jia et al., 2018). We performed qRT-PCR to determine the transcription levels of *AtMYB58*, *AtMYB4*, and *At4CL1* in WT *Arabidopsis* and CsMYB85-OE transgenic plants, and accordingly observed that *AtMYB58*, *AtMYB4*, and *At4CL1* expression levels were upregulated in CsMYB85-OE transgenic plants (Figure 8B) and that the lignin content in these transgenic plants was 188% higher than that in the WT plants (Figure 8C).

## Discussion

In this study, we identified and isolated the MYB transcription factor *CsMYB85* from *Citrus sinensis*. Sequence analysis based on alignment and phylogenetic tree construction indicated that the function of *CsMYB85* is conserved with its orthologs in other plant species (Figure 1). *CsMYB85* expression was found to increase significantly during the progression of juice sac granulation (Figure 2), and we determined that CsMYB85 localizes in the nuclei of transformed *N. benthamiana* cells and has transcriptional activity in yeast (Figure 3). Furthermore, yeast one-hybrid and transcriptional activation assays indicated that CsMYB85 regulates *CsMYB330* expression by binding the *CsMYB330* promoter (Figure 4). In addition, *CsMYB85* overexpression in juice sacs and *A. thaliana* revealed that CsMYB85 regulates lignin biosynthesis (Figure 5, 6), and therefore we surmise that CsMYB85 may regulate lignin biosynthesis during fruit juice sac granulation in *C. sinensis*.

Previous studies have shown that cell wall thickening and lignin deposition occur during juice sac granulation (Burns and Achor, 1989; Shomer et al., 1989), and that the levels of lignin, pectin, hemicellulose, and cellulose all increase in the cell walls of granulating fruit juice sacs (Shomer et al., 1989; Zhang et al., 2016). To date, however, little attention has been focused on the identity and role of the transcription factors involved in this process. In a previous paper, we reported that the transcription factors CsMYB330 and CsMYB308 finely regulate *Cs4CL1* expression and participate in citrus fruit juice sac lignification (Jia et al., 2018). We also established that CsMYB85 does not bind to the promoters of lignin synthesis-related genes, but does bind to the promoter of the CsMYB330 transcription factor, via which it is assumed to indirectly regulate citrus fruit juice sac lignification.

Transcription factors play prominent roles in regulating gene expression (Taylorteeples et al., 2015). In *Arabidopsis* and other plants, MYB and NAC family members, including AtMYB85, AtMYB58, AtMYB63, NST1, and NST2, regulate secondary cell wall biosynthesis (Zhou et al., 2009; Zhong et al., 2011; Taylorteeples et al., 2015). Lignin has been found to be ectopically deposited in stem epidermal and cortical cells of plants overexpressing *AtMYB85*, whereas no corresponding ectopic deposition of cellulose or xylan deposition was observed in these cells. Therefore, it is assumed that overexpression of *AtMYB85* promotes the exclusive induction of the lignin biosynthetic pathway. Previously, it was found that *AtMYB85* specifically induces GUS expression under control of the *At4CL1* promoter (Zhong et al., 2008), and in the present study, we found that lignin content was increased and *At4CL1* expression was significantly upregulated in *Arabidopsis* plants overexpressing *CsMYB85* (Figure 8). Given that yeast one-hybrid assays indicated that CsMYB85 does not bind to the Cs4CL1 promoter, we propose that CsMYB85 regulates *Cs4CL1* expression via other transcription factors such as CsMYB330 or CsMYB308.

Analysis of the promoters of lignin synthesis-related genes revealed that the AC *cis*-acting elements of these promoters are recognized by MYB transcription factors and have widespread occurrence (Rogers and Campbell, 2004). Numerous studies have found that *Arabidopsis* AtMYB4, AtMYB58, and AtMYB63, *C. sinensis* CsMYB330 and CsMYB308, *E. japonica* EjMYB1 and EjMYB2, and maize ZmMYB31 transcription factors can bind to AC elements, and thereby regulate gene expression (Zhao et al., 2007; Zhou et al., 2009; Fornalé et al., 2010; Xu et al., 2014; Jia et al., 2018). In the present study, we determined that the CsMYB85 transcription factor binds to the promotor of *CsMYB330* *in vitro* (Figure 4). However, that fact that the *CsMYB330* promoter lacks AC elements indicates that some MYB transcription factors recognize sequences other than AC elements and are thus functionally differentiated within their family. Although the *Cs4CL1* promoter contains AC elements (Jia et al., 2018) and CsMYB85 induces *Cs4CL1* expression (Figure 7B), CsMYB85 cannot physically interact with the *Cs4CL1* promoter (Figure 4), and thus it must be assumed that CsMYB85 indirectly regulate *Cs4CL1* expression. Similar findings have been reported for the AP2/ERF transcription factor family. For example, EjAP2-1 has been shown to induce *E. japonica* fruit lignification by interacting with EjMYB transcription factors (Zeng et al., 2015). In *Arabidopsis*, the AP2 transcription factor AtSHINE (AtSHN) is associated with increased cellulose levels and decreases lignin content by interacting with the promoters of putative rice NAC and MYB transcription factor genes, thereby regulating their expression. Certain transcription factors, including NST1/2/SND1, MYB20/43, MYB58/63, and VND4/5/6, are known to be secondary wall regulators (Ambavaram et al., 2011), and thus EjAP2-1 and AtSHINE are assumed to regulate lignification via different mechanisms. Similarly, our findings revealing that CsMYB85 binds to the CsMYB330 promoter and interacts with CsMYB308 indicate that CsMYB85 regulates citrus fruit lignification through different routes.

Figure 7, 8 show that the expression levels of *CsMYB308* and *AtMYB4* were significantly upregulated in response to overexpression of *CsMYB85*. However, Figure 4 indicates that CsMYB85 does not bind to the CsMYB308 promoter, although Figure 5, 6 reveal that CsMYB85 does interact with CsMYB308 in yeast and tobacco leaves. In a previous study, we found that *CsMYB308* expression levels were downregulated during juice sac lignification and identified CsMYB308 as a transcriptional repressor in citrus fruit juice sac lignification (Jia et al., 2018). Given that, in the present study, we found that *CsMYB308* expression levels were significantly upregulated in response to overexpression of *CsMYB85* (Figure 7), we speculate that citrus may inhibit fruit juice sac lignification in response to stress. In *Citrus maxima* “Hongrou-miyou,” *CmMYB330* expression levels were highest 208 days after flowering and thus CmMYB330 may have strongly inhibited fruit juice sac lignification during this period. It is therefore conceivable that CmMYB330 inhibits the onset of granulation and impede its progress (Yang et al., 2017a).

## Conclusion

**FIGURE 9:**
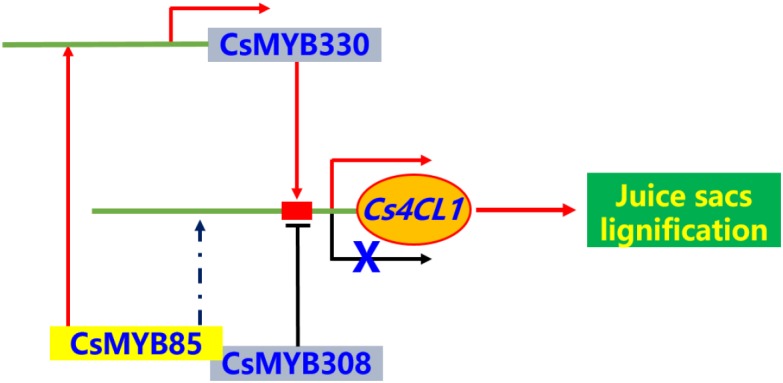
The brief model for regulatory network of *Citrus sinensis* fruit juice sacs lignification. The MYB transcription factors can activate or inhibit the expression of lignin biosynthesis related genes in *Citrus sinensis* fruit juice sacs. The *Cs4CL1* gene directly involved in lignin biosynthesis. The transcription factors, CsMYB330 and CsMYB330, regulated expression of *Cs4CL1* gene, directly. The CsMYB85 transcription factor can interactive with CsMYB308 and regulated expression of *CsMYB330* gene, and indirectly regulated expression of *Cs4CL1* gene.

In the present study, we identified and isolated the transcription factor CsMYB85, a novel regulator of *Citrus* fruit juice sac lignification. The results showed that CsMYB85 regulates *Citrus* fruit juice sac lignification via different mechanisms. CsMYB85 binds the promoter of *CsMYB330*, which is a lignin biosynthetic-related regulator. In this way, it induces both *CsMYB330* and its downstream genes, including *Cs4CL1.* These observations suggest that the CsMYB85 has regulatory functions similar to those of its transcription factor homolog in *Arabidopsis*. Together with the findings from previous studies have revealed a regulatory network is involved in the transcriptional activation of *Citrus sinensis* fruit juice sacs lignification (Figure 9). We believe that the findings of the present study contribute to establishing the functional classification and elucidating the regulatory mechanisms of MYB transcription factors in postharvest citrus fruit lignification.

## Author Contributions

NiJ, FX, and BF designed the study. NiJ, JiqL, PT, YS, YL, JiaL, JS, YH, JL, NuJ, ML, and KM performed the experiments. NiJ, FX, and BF wrote the manuscript. All authors have read and approved the manuscript.

## Conflict of Interest Statement

The authors declare that the research was conducted in the absence of any commercial or financial relationships that could be construed as a potential conflict of interest.
