# Supplementary material for: Citrus sinensis MYB Transcription Factor CsMYB85 Induce Fruit Juice Sac Lignification Through Interaction With Other CsMYB Transcription Factors
Source: Front Plant Sci. 2019 Feb 25;10:213. doi: 10.3389/fpls.2019.00213 (PMC6401657; doi:10.3389/fpls.2019.00213)
Supplement: Supplementary file 1 [file Table_1.docx]

**Supplementary Table 1** Primers used for the qRT-PCR analysis.

| Primer name | Primer sequences |
| --- | --- |
| CsMYB330-15 | 5'- GGG TTT AGT GAA CCT TAT GAT GTT GC -3' |
| CsMYB330-14 | 5'- CTC GCA AAC GGA ACT TTG TAG TGA AGC -3' |
| CsMYB308-15 | 5'- CTA CTA CTG CTG CTC CTG CTG ATG TG -3' |
| CsMYB308-14 | 5'- TTT CAT CTC CAA GCT TCT GTA ATC CAA AAC -3' |
| CsMYB85-12 | 5'- GGG AAT CGA TCC TGT CAC TCA TGA GCC -3' |
| CsMYB85-11 | 5'- GTG CCT GCC ACC CAC CTC CAA ATT G -3' |
| Cs4CL1-9 | 5'- GCA TAA GAG GAG ATC AAA TCA TGA CAG -3' |
| Cs4CL1-10 | 5'- CAG CCG CTA ATT TAG CTC TTA ATT CC -3' |
| CsActin-1 | 5'- GGT CGT ACA ACT GGT ATT GTG CTG G -3' |
| CsActin-2 | 5'- CAT TTC CTG TGG ACA ATG GAT GGA C -3' |
| AT4CL1-RT1 | 5'- GTT GCT GTT GTC GCA ATG AAA GAA GAA G -3' |
| AT4CL1-RT2 | 5'- CAC AAT CCA TTT GCT AGT TTT GCC C -3' |
| ATMYB58-RT1 | 5'- GAT GGT TCA GAC TCA TTC CAA CAA CC -3' |
| ATMYB58-RT2 | 5'- GTA TGA GGA GCT CGT AAC TCT CCA AG -3' |
| ATMYB4-RT1 | 5'- GTC AAC AAC GCC ACG TTG TTT CAA G -3' |
| ATMYB4-RT2 | 5'- CTT CGA AAG CCC AAA AGA GAA GTG G -3' |
| AtUBQ-F | 5'- AGT CCA CCC TTC ATC TTG TTC TC -3' |
| AtUBQ-R | 5'- GTC AGC CAA AGT TCT TCC ATC T -3' |

**Supplementary Table 2** Primers used for the vector construction.

| Primer name | Primer sequences |
| --- | --- |
| CsMYB85-3 | 5'- CCT ACT AGT ATG GGC AGG CAA CCT TGC TGT GAC -3' |
| CsMYB85-4 | 5'- CCT ACG CGT GTG CCT GCC ACC CAC CTC CAA ATT G -3' |
| CsMYB85-9 | 5'- CCT CAG ATG GGC AGG CAA CCT TGC TGT GAC -3' |
| CsMYB85-10 | 5'- CCT GGA TCC CTA GTG CCT GCC ACC CAC CTC CAA ATT G -3' |
| CsMYB85-7 | 5'- CCT GGA TCC ATG GGC AGG CAA CCT TGC TGT GAC -3' |
| CsMYB85-13 | 5'- CCT CTG CAG CTA GTG CCT GCC ACC CAC CTC CAA ATT G -3' |
| CsMYB85-14 | 5'- CCT GGT ACC ATG GGC AGG CAA CCT TGC TGT GAC -3' |
| CsMYB85-15 | 5'- CCT CTC GAG GTG CCT GCC ACC CAC CTC CAA ATT G -3' |
| CsMYB330-11 | 5'- CAA ACT AAT AGA AGT GCC CAC AAG TTT TG -3 |
| CsMYB330-12 | 5'- CCT ACG CGT CAG ACT CCC AAC TGA CTG AAG AC -3' |
| CsMYB330-18 | 5'- CCT GAC GTC CAA ACT AAT AGA AGT GCC CAC AAG TTT TG -3' |
| CsMYB330-19 | 5'- CCT CTC GAG CAG ACT CCC AAC TGA CTG AAG AC -3' |
| CsMYB308-11 | 5'- GAG TAT CTT AGG GAA CCA TCG GCT AAG -3' |
| CsMYB308-12 | 5'- CCT ACG CGT TTT CCT ATT ATA TCC TGT TCT CGG AAG C -3' |
| CsMYB308-7 | 5'- CCT GGA TCC GAG TAT CTT AGG GAA CCA TCG GCT AAG -3' |
| CsMYB308-17 | 5'- CCT CTC GAG TTT CAT CTC CAA GCT TCT GTA ATC CAA AAC -3' |
